# Severe Bradycardia Leading to Hemodynamic Instability Associated with Remdesivir Use in a Patient with COVID-19 Pneumonia

**DOI:** 10.1155/2022/8807957

**Published:** 2022-10-11

**Authors:** Bhargavi Donepudi, Shikhar Agarwal, Lokendra Thakur

**Affiliations:** ^1^Department of Critical Care Medicine, Geisinger Medical Center, 100N Academy Ave, Danville, PA, USA; ^2^Department of Cardiology, Geisinger Medical Center, 100N Academy Ave, Danville, PA, USA

## Abstract

Remdesivir (RDV) is an approved treatment for hospitalized patients with severe acute respiratory syndrome coronavirus 2 (SARS-CoV-2) infection. There is limited literature on the cardiac adverse effects of RDV. We report a case of a patient who developed hemodynamically unstable bradycardia after the initiation of RDV that resolved after discontinuing RDV.

## 1. Introduction

Since the emergence of COVID-19 pandemic, RDV has been one of the leading antiviral medications that is being used worldwide to treat SARS-CoV-2 infection. It was FDA approved in October of 2020. ACTT-1 trial has demonstrated that RDV shortens the time to clinical recovery if it is used within the first 7-10 days of symptom onset [1]. Solidarity and Discovery trials did not demonstrate benefit of RDV with regard to mortality, initiation of mechanical ventilation, time to clinical recovery, or duration of hospital stay [2, 3]. Most hospital guidelines continue to recommend RDV use in the treatment of patients hospitalized with COVID-19 infection in a patient who require oxygen supplementation for hypoxia. Therefore, it is important for us to know the adverse reactions of RDV, most importantly its cardiac side effects which can lead to significant hemodynamic effects like in our patient.

## 2. Case Presentation

We are presenting a 43-year-old female with no significant past medical history (PMH) who was presented to the emergency department with typical symptoms of SARS-CoV-2 infection for the last five days including high-grade fever, cough, shortness of breath, abdominal pain, loss of appetite, and several episodes of vomiting. At the time of presentation, she had not received vaccination against COVID-19. SARS-CoV-2 was confirmed using polymerase chain reaction (PCR). On physical exam, she had dry mucous membranes and diffuse coarse breath sounds with decreased bibasilar breath sounds. On presentation, she was noted to have an oral temperature of 101.5 F, blood pressure of 80/50 mmHg, heart rate (HR) of 69 beats/min, and an oxygen saturation of 99% on room air. Her body mass index (BMI) on presentation was calculated to be 31 kg/m^2^. She received a total of 5 L normal saline and was started on intravenous norepinephrine (2 mcg/min) to maintain mean arterial pressure > 65 mmHg. She was admitted to critical care unit for further management as she continued to require vasopressor support.

## 3. Investigations

Labs on admission revealed mild range pancytopenia (white blood cell count: 1,870 per microliter (reference range: 4,000-10,000/microliter), hemoglobin: 9.1 grams/deciliter (reference range: 11.4-14.4 grams/deciliter), and platelet count: 1,25,000 per microliter (reference range: 1,50,000-4,00,000/microliter)), elevated C-reactive protein levels (12 milligrams/liter, reference range: less than 10 milligrams/liter) with normal creatinine, hepatic function panel, lactate, ferritin, and troponin levels. Chest X-ray and computed tomography showed diffuse bilateral consolidative and ground glass opacities consistent with COVID-19 pneumonia with no clear evidence of superimposed bacterial infection.

**Figure 1:**
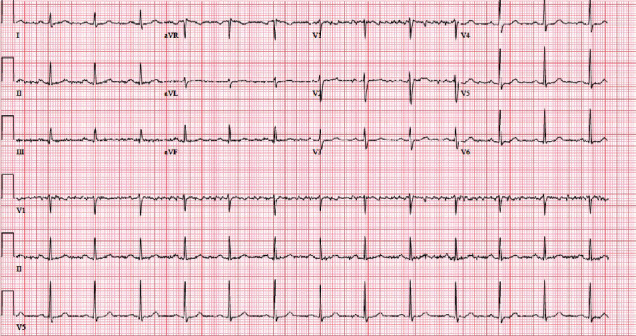
Normal ECG at the time of admission.

Electrocardiogram (ECG) on admission showed normal sinus rhythm with ventricular rate of 79 beats/min, normal PR interval of 176, and QTc interval of 408 milliseconds (Figure 1).

## 4. Differential Diagnosis

COVID-19 induced sinus node dysfunction, acute coronary syndrome, heart block, and other drug effects.

## 5. Management

**Figure 2:**
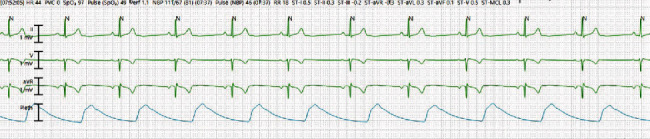
Telemetry strips showing bradycardia after the initiation of RDV (day one).

On admission to critical care unit, patient was started on dexamethasone 6 mg IV daily and empiric broad spectrum antibiotics including vancomycin and cefepime (which were later discontinued after blood cultures were found negative). Based on the current dosing guidelines, RDV was initiated (loading dose of 200 mg on the day of admission, which was later followed by 100 mg for 4 more days) [1]. Immediately after the loading dose of the RDV infusion, patient was noted to develop bradycardia and worsening hypotension requiring escalation of the norepinephrine infusion up to 5 mcg/min. She continued to be bradycardic to as low as 41 beats/min on telemetry monitoring during which she endorsed dizziness and chest pressure (Figure 2). Dopamine infusion was started at 5 mcg/kg/min which was slowly titrated up to 15 mcg/kg/min to maintain adequate HR and target mean arterial pressure (MAP) > 65 mmHg. Norepinephrine was subsequently stopped.

**Figure 3:**
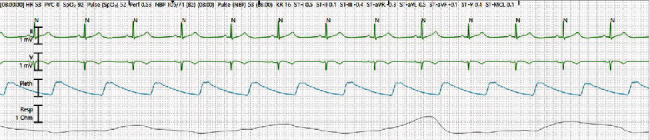
Telemetry strips showing bradycardia after the initiation of RDV (day two).

**Figure 4:**
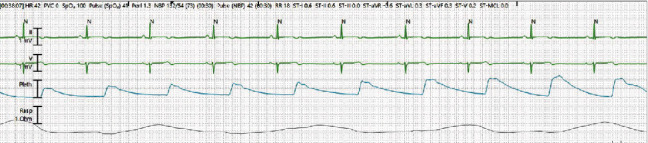
Telemetry strips showing bradycardia after the initiation of RDV (day four).

Throughout the five-day course of RDV, patient's HR ranged between 40 and 60 beats/min as noted on continuous telemetry monitoring (Figures 3 and 4) requiring dopamine infusion at a rate of 15 mcg/kg/min with highest requirement being 16.5 mcg/kg/min to maintain MAP > 65 mmHg. Echocardiogram was obtained which showed normal ejection fraction of 60% with no wall motion or valvular abnormalities. Patient's bradycardia and hypotension resolved within 30 hours of the last dose of RDV. We were able to wean off both norepinephrine and dopamine infusion. She remained in normal sinus rhythm with normotension, and patient was discharged home in stable condition.

## 6. Discussion

RDV is the most common antiviral medication used in the treatment of SARS-CoV-2 infection. We have very limited data on its cardiac adverse effects. Arrhythmias including atrial fibrillation and supraventricular tachycardia, hypotension, and rarely cardiac arrest were reported in recently conducted largest randomized controlled trials on RDV, ACTT-1 (Adaptive COVID-19 Treatment Trial), Solidarity, and Discovery trials [1–3]. No data have been reported on bradycardia. With the increasing use of RDV, there have been handful of case reports on RDV-induced sinus bradycardia. To the best of our knowledge, this is only a second description on RDV-induced hemodynamically unstable symptomatic bradycardia that required vasopressor support which resolved after discontinuation of RDV [4–7].

In the year 2021, an observational study was conducted using VigiBase which is a World Health Organization (WHO) Global Database of Individual Case Safety Reports (ICSRs) to investigate the association between RDV use and increased risk of bradycardia. They conducted disproportionality analyses to assess the increased risk of reporting bradycardia (reporting odds ratio (ROR) with 95% CI) with RDV compared to other drugs that were used in the treatment of COVID-19 disease. They found that 31% of the cardiac events that were reported included bradycardia out of which 17% were thought to be fatal [8]. Later that year, another study was published by Singh and Kamath on adverse event reports submitted to the United States Food and Drug Administration Adverse Event Reporting System (FAERS) associated with RDV use for COVID-19 using real-world data which also showed disproportionately high report of bradycardia, cardiac arrest, and death with RDV use [9].

RDV is a nucleoside analogue prodrug which is known to inhibit viral RNA-dependent RNA polymerase (RdRP) that plays a key role in viral replication. It has been observed in vitro that RDV causes similar inhibition of RdRP among all coronaviruses including SARS-CoV-2.

RDV is intracellularly metabolized to its active triphosphate (RTP) form which resembles adenosine triphosphate (ATP). RTP competes with ATP to be incorporated into RdRP and has higher affinity towards viral RdRP compared to ATP. Once incorporated, it causes delayed chain termination of RNA synthesis and thus inhibits viral replication [10]. Half-life elimination of RDV is ~1 hour and its metabolite is ~27 hrs.

The mechanism underlying the RDV-induced bradycardia is not well known. It is believed that due to the close structural resemblance between the RTP and the ATP, RTP might also exert similar negative chronotropic and dromotropic effects on the hearts conducting system that ATP and its metabolite adenosine exhibits. Another possible mechanism is drug-induced cardiotoxicity. Despite RDV strong affinity towards viral RNA polymerase, it can sometimes cross react with human RNA polymerase leading to mitochondrial dysfunction causing cardiotoxicity [4, 5, 8].

Additionally, severe COVID-19 infection has been associated with causing cardiac abnormalities including myocarditis, myocardial infarction, arrhythmias, cardiac arrest, and sinus node dysfunction leading to bradycardia. The leading mechanisms behind SARS-CoV-2-induced cardiac abnormalities include direct cardiac injury from the infection, severe hypoxia, and cytokine storm with intense inflammatory reaction affecting the cardiac pacemaker cells [11]. However, in our case, the patient did not have any significant lab findings (near normal C-reactive protein) or respiratory symptoms with severe hypoxia to demonstrate severe infection, and patient's hemodynamically unstable bradycardia resolved within 24-48 hours after discontinuing RDV. The causality assessment score for suspected adverse drug reactions (ADRs) was performed using the Naranjo probability scale which determines the likelihood of whether the ADR is due to the drug rather than the other causes [12]. A score of 5 was obtained which supports our suspicion that this reaction was a probable ADR from RDV use.

## 7. Conclusion

RDV is being used worldwide as a standard of care for the treatment of COVID-19 infection. Though none of the major clinical trials have demonstrated bradycardia as a safety concern, sinus bradycardia with and without hemodynamic instability is being increasingly reported. Obtaining baseline ECG and continuous cardiac monitoring throughout the RDV use is recommended to avoid fatal outcomes. Dopamine can be used in patients with hemodynamically unstable sinus bradycardia to support them through the course of RDV. More studies are needed to further investigate the cardiac adverse effects of RDV.
